# Study of the Preparation and Properties of Chemically Modified Materials Based on Rapeseed Meal

**DOI:** 10.3390/biom14080982

**Published:** 2024-08-10

**Authors:** Sara Aquilia, Luca Rosi, Michele Pinna, Sabrina Bianchi, Walter Giurlani, Marco Bonechi, Francesco Ciardelli, Anna Maria Papini, Claudia Bello

**Affiliations:** 1 Interdepartmental Research Unit of Peptide and Protein Chemistry and Biology, University of Florence, Via della Lastruccia 13, I-50019 Sesto Fiorentino, Italy; sara.aquilia@unifi.it (S.A.); claudia.bello@unifi.it (C.B.); 2 Department of Chemistry “Ugo Schiff”, University of Florence, Via della Lastruccia 13, I-50019 Sesto Fiorentino, Italy; luca.rosi@unifi.it (L.R.); walter.giurlani@unifi.it (W.G.); marco.bonechi@unifi.it (M.B.); 3 Spin-PET S.r.l., Viale R. Piaggio 32, I-56025 Pontedera, Italy; pinna@spinpet.it (M.P.); bianchi@spinpet.it (S.B.)

**Keywords:** rapeseed meal-based material, protein-based biopolymers, renewable biomaterials

## Abstract

In recent years, there has been increasing interest in developing novel materials based on natural biopolymers as a renewable alternative to petroleum-based plastics. The availability of proteins derived from agricultural by-products, along with their favourable properties, has fostered a renewed interest in protein-based materials, promoting research in innovative technologies. In this study, we propose the use of rapeseed protein-rich meal as the main ingredient for the preparation of novel sustainable materials combining excellent environmental properties such as biodegradability and renewability. The application of sustainable products in the present high-tech society requires the modification of the basic native properties of these natural compounds. The original route proposed in this paper consists of preparation via the compression moulding of flexible biomaterials stabilized by crosslinkers/chain extenders. An investigation of the effects of different denaturing and disulfide bond reducing agents, crosslinkers, and preparation conditions on the material mechanical behaviour demonstrated that the novel materials have appreciable strength and stiffness. The results show the potential of utilizing full meal from vegetable by-products to prepare protein-based materials with guaranteed ecofriendly characteristics and mechanical properties adequate for specific structural applications.

## 1. Introduction

Polymeric organic materials are extensively used across various sectors in modern society. The remarkable properties and versatility of polymers, coupled with their cost-effectiveness, have resulted in their widespread usage. Nonetheless, the unregulated disposal of the huge amount of plastic waste generated has given rise to one of the most pressing environmental challenges of our era [1,2,3], which mainly concerns packaging and low-performance, short-life products.

Consequently, there is a growing interest in the development of environmentally friendly and biodegradable materials from renewable resources. This effort aims to replace the use of petroleum-based materials in sustainable applications, which raise significant concerns regarding pollution and sustainability [4]. However, at present, bioplastics and in general bioderived organic materials constitute less than one percent of the annual production of plastic, which exceeds 390 million tons [5]. Transition to a circular economy model is also necessary for the development of innovative strategies to optimize resource utilization and eliminate the concept of waste throughout the entire supply chain [6,7,8,9]. Bio-related plastics and organic materials derived from coproducts or waste materials offer the advantage of exploiting pre-existing, and often underutilized, resources. Furthermore, their utilization serves to mitigate disposal costs and to reduce environmental impacts [10].

Proteins from the agri-food industry are a good alternative to petrochemical-based polymers. Rapeseed is a crucial oilseed crop, ranking second only to soybean, with Canada and United States being the top producers, while Europe accounts for almost 25% of the global production [11,12]. Rapeseed is manly employed for oil production in the food and feed industry, as well as for biodiesel preparation. The residual product generated by oil extraction is the rapeseed meal, also called canola meal, which is collected as a by-product after oil pressing and subsequent solvent extraction [12]. Depending on the growth conditions, harvest methods, and processing techniques, the protein content in rapeseed meals may vary from 35% to 40% [13]. Cruciferin (12S globulin) and napin (2S napin) represent 60% and 20% of the total rapeseed crude protein, respectively. Cruciferin is a high-molecular-weight protein formed by six subunits each containing a heavy α-chain (30 KDa) and a light β-chain (20 KDa), linked by both disulfide bonds and non-covalent interactions. Napin is instead composed of one small (4–6 kDa) and one large (10–12 kDa) polypeptide chain that are held together by disulfide bonds [14,15].

Although rapeseed meal possesses significant nutritional potential and contains value-added compounds [16,17], its application in the food and feed sectors is limited by the presence of antinutritional compounds, such as glucosinolates, erucic acid, phytates, and phenols, which negatively affect the palatability and digestibility of the meal [12]. In recent years, because of its high storage protein content and low cost, the exploitation of canola meal for the production of cosmetics and bioplastic materials has emerged [13]. Rapeseed proteins offer indeed a promising ecofriendly alternative for the production of technical products such as polymers, coatings, adhesives, detergents and lubricants [18]. The development of a fully natural and potentially biodegradable protein-based material with acceptable mechanical properties could efficiently decrease the societal demand for fossil-based polymers in several applications [19].

However, the application of proteins, including proteins from rapeseeds, as bio-based polymers is limited by certain drawbacks, including slow biodegradability, limited mechanical properties and high water permeability [7,20,21,22]. Additives, such as plasticizers, are commonly integrated into the biopolymers to improve these limitations. Plasticizers break inter- and intra-molecular hydrogen bonds, resulting in the enhanced mobility of polymer chains, greater spacing between polymer molecules, and a reduction in the proportion of crystalline regions compared to amorphous regions. Glycerol and water are common plasticizers in protein-based materials, because of their low molecular mass and ability to interact with polar residues [23,24,25]. Additionally, crosslinkers are used to enhance the mechanical strength, the thermal and chemical stability, and to decrease the swelling properties of polymeric and biopolymeric materials [26,27]. Nevertheless, high crosslinking might lead to a decrease in the biodegradability of the material. Therefore, using eco-compatible crosslinkers and optimizing the amount of crosslinker in the material enable the exploitation of its favourable effects while minimizing the drawbacks.

The production of edible films and bio-composites from agricultural process by-products has been already reported [28,29,30,31]. However, the development of commercially suitable industrial products from raw rapeseed meal is very limited [19,23,32] and the studies involving rapeseed biomass are mainly focused on protein isolate-based bioplastics and on wet casting techniques [33,34,35,36] (Table 1).

On the other hand, our study aims to utilize the rapeseed meals as a matrix for the development of a cost-effective biodegradable composite for possible future industrial applications. Therefore, we describe herein an original route for the preparation of flexible biomaterials based on raw, protein-rich rapeseed meal stabilized by modulated crosslinking with selected epoxides, using compression moulding. Moreover, we investigated the effects of different denaturing and disulfide bond-reducing agents, cross-linkers, and preparation conditions on the mechanical behaviour. The thermal properties were also studied through TGA, thus showing the potential use of our approach in the preparation of novel biocompatible materials from vegetable waste.

## 2. Materials and Methods

### 2.1. Materials

The rapeseed meal (RM) was generously supplied by Italcol S.p.A. (Castelfiorentino, Italy). Rennet casein was provided by Fontana Enzo s.r.l. (Sarmato, Italy). Glycerol, poly(ethylene glycol) diglycidyl ether, neopentyl glycol diglycidyl ether, epoxidized soybean oil, sodium sulfite (Na_2_SO_3_), sodium dodecyl sulfate (SDS), urea, guanidinium chloride, and anhydrous KBr were purchased from Sigma-Aldrich (St. Louis, MO, USA). They were of analytical or reagent grade and were employed without additional purification.

### 2.2. Rapeseed Meal-Based (RM) Material Preparation

The rapeseed meal was dry milled at 1800 rpm for 15 min at room temperature. The resulting powder was placed in an oven overnight at 50 °C to remove moisture. Next, 40 g of rapeseed meal (53% *w*/*w* total) was mixed with 10 g of a protein-binding agent (Rennet casein, 13 *w*/*w*), 20 g of glycerol (27% *w*/*w*), 5 g of distilled water (7% *w*/*w*), and 2.5 g of Poly(ethylene glycol) diglycidyl ether (2 × mol lysine-based), using a blender blade for 15 min to obtain a homogeneous blend. Glycerol was used as a plasticizer, and Poly(ethylene glycol) diglycidyl ether was employed as a cross-linker. Subsequently, the mixture was heated to 150 °C for 15 min and pressed using a flat-bed press (CAMPANA model: PRESSA PM20/200) at 150 °C for 20 min at 250 Bar (mould dimensions: 8 cm × 16 cm × 1.5 mm). The resulting material (RM, Table 2) was then cooled to room temperature and stored in a desiccator with 75% humidity control.

Modified rapeseed meal specimens (see Table 2 for detailed composition) were prepared following the above-described procedure, except for the substitution of the distilled water with an aqueous solution of Na_2_SO_3_ (10% *w*/*w*), Na_2_SO_3_ (20% *w*/*w*), Urea (2 M), Guanidine (1 M), or SDS (3% *w*/*w*).

### 2.3. Mechanical Properties

After pressing, the rapeseed meal specimens were kept at 25 °C and 75% RH for 24 h, and then cut into three or four standard dumbbell-shaped moulds, each measuring 60 mm (length) × 5 mm (width) × 1.3 mm (thickness). The mechanical properties were evaluated using a Shimadzu instrument (Model AGS-X 5 KN, Milano, Italy) following the Standard Test Method for Tensile Properties of Plastics (ASTM D638-91). The testing speed was set at 5 mm/min. The tensile strength, elongation at break (%), and elastic modulus were determined based on measurements taken from at least three replicates.

### 2.4. Fourier-Transform Infrared Spectroscopy

Fourier-Transform Infrared Spectroscopy (FT-IR) analysis of the specimens was conducted in transmittance mode using a Jasco FT-IR-4200 instrument (Cremella, Italy). Prior to analysis, the materials were dried in an oven at 60 °C for 48 h. Subsequently, the dried samples were milled into powder using a mortar and pestle and blended with anhydrous KBr at a 1:50 ratio. The resulting mixture was then pressed to form transparent pellets. Spectra were collected over the range of 400 to 4000 cm^−1^ with 32 scans at a resolution of 4 cm^−1^. The spectra were further analyzed using Origin Pro 2021 by baseline subtraction and normalization in the range 1:100.

### 2.5. Thermogravimetric Analysis

The thermal stability of the protein-based materials was assessed via thermogravimetric analysis (TGA) conducted utilizing a Perkin Elmer 4000 instrument (Waltham, MA, USA). The samples were subjected to controlled heating under a continuous flow of nitrogen (30 mL/min) from room temperature to 815 °C at a programmed ramping rate of 10 °C/min.

### 2.6. Scanning Electron Microscopy

Scanning electron microscopy (SEM) analysis was performed with a variable pressure Hitachi SU3800 (Hitachi High-Tech, Tokyo, Japan) equipped with a Ultim Max 40 silicon drift EDS detector and AZtecLive software 6.1 (Oxford Instruments NanoAnalysis, Abingdon, UK). The measurements were performed at various magnifications with an accelerating voltage of 15 kV. Before the SEM analysis, the samples were metallized using a SC7640 Polaron Sputter Coater (Quorum Technologies, Laughton, UK) with a SC510-314B gold/palladium target. Both the surface and the section of the samples were analyzed. For sectioning, we opted for cryofracture: the samples were immersed for approximately one minute in liquid nitrogen to ensure that all the material reached the same temperature, and then it was split in two using two forceps while keeping the polymer immersed in liquid nitrogen.

## 3. Results and Discussion

Preliminary tests to assess the behavior of canola meal in hot pressing were carried out, and optimized temperature (150 °C) and pressure (250 bar) conditions were determined. Afterwards, the influence of the method used for blend preparation and of the addition of plasticizers and cross-linkers was evaluated. The best results were obtained using a blender blade as the mixing method, water and glycerol as plasticizers, and poly(ethylene glycol) diglycidyl ether (Epoxy-PEG) as a cross-linker.

Glycerol, an environmentally friendly, effective plasticizer for biopolymer-based materials [37], in particular those derived from proteins [38], was used in the same quantity as in applications reported in the literature [39,40,41]. Epoxy-PEG, a hydrophilic epoxidized compound, which can be also obtained from natural resources, was used in an amount based on the moles of Lys (2.86% of the full amino acid content) present in the meal proteins [42]. The epoxy groups exhibit high reactivity with the amine and carboxyl functional groups present on the side chains of the protein amino acids. Notably, even at 70 °C, the amine and epoxy groups demonstrate a significant reaction rate [19]. Further significant improvements were obtained when adding Rennet casein [43,44] and canola meal/casein in a 4:1 ratio to the preparation. The use of Casein derived from surplus milk not intended for the food industry constitutes a deliberate effort to optimize the use of environmental resources and reduce the ecological impact on our planet.

Those preliminary tests demonstrated that raw canola meal can be used in heat pressing processes to produce a compact material with promising mechanical properties even if still not at the level of fossil plastic polymers.

### 3.1. Influence of Different Crosslinkers on the Tensile Properties of the Specimens

Starting from the results obtained from the preliminary tests, the effect of three different epoxydic crosslinkers on the properties of our material was evaluated. We thus compared the hydrophilic Epoxy-PEG with epoxidized soybean oil, a biobased hydrophobic crosslinking agent already used as stabilizer and plasticizer in PVC and bio-based materials [45,46], and neopentyl glycol diglycidyl ether. The latter is hydrophobic in nature, with a smaller steric size and lower molecular weight than the other two. This may allow for better diffusion within the blend, compared to the epoxidized soybean oil [46,47,4]. The Young’s modulus and the stress at fracture of samples RM-ES and RM-NE decreased substantially, as reported in Table 3. The particularly low elongation is probably primarily due to the structural microfracture of the materials deriving from the not complete and uniform diffusion of the crosslinker in the protein-rich matrix and the heterogeneous chemical nature of the matrix itself. Moreover, only the material prepared using epoxidized soybean oil (RM-ES) and neopentyl glycol diglycidyl ether (RM-NE) showed an exudate on the surface a few hours after preparation, probably due to the migration of the non-reacted crosslinker.

### 3.2. Effects of Disulfide Bond Reducing Agents and Denaturant

Disulfide-bond-reducing or/and denaturing agents in different percentages were added to the blend to improve the mechanical properties of the material.

**Figure 1:**
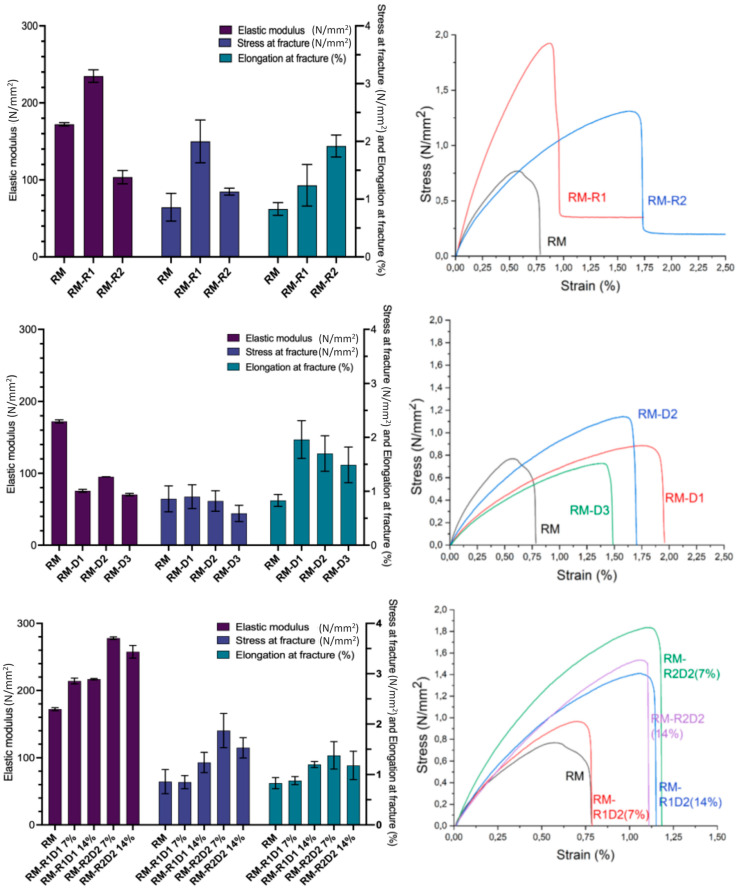
Histogram of tensile properties (**left**) and stress/strain diagram (**right**) of meal/casein/water/glycerol blends without or with the addition of Na_2_SO_3_ and/or a denaturing agent (for specimens’ composition refers to Table 2).

The tensile strength and Young’s modulus increased with the addition of a 10% *w*/*v* aqueous solution of Na_2_SO_3_ (RM-R1, Table 4) when compared to the unmodified RM material. However, the material made with a 20% *w*/*v* aqueous solution of Na_2_SO_3_ showed a comparable tensile strength to the RM material, but with a higher elastic modulus and elongation at fracture (Figure 1 and Table 4). The mechanical properties of the material exhibited a strong correlation with their molecular structures and constituent components. Na_2_SO_3_ acts as a reducing agent for the disulfide bonds within the proteins, thereby inducing protein denaturation. Certain functional groups, such as amino, carboxylic, and sulfhydryl groups, exhibit relatively high reactivity and can participate in reactions with both Epoxy-PEG and other functional groups, facilitating the formation of cross-links, particularly under the elevated temperatures of processing.

Modified RM materials such as RM-R1 and RM-R2, characterized by a high degree of denaturation, tend to develop a greater number of entanglements and crosslinks, resulting in enhanced mechanical strength. At higher concentrations, Na_2_SO_3_ primarily serves as a plasticizer, increasing the mobility of the protein chains. However, the elongation at fracture (Figure 1 and Table 4) remains low, thus suggesting that the mobility is limited to small domains inside the material surrounded and confined by densely crosslinked species. The use of TCEP (tris(2-carboxyethyl)phosphine) as an alternative reducing agent to sodium sulfite was not effective, as the tensile properties of the material worsened dramatically when a 10% *w*/*v* aqueous solution of TCEP (7% *w*/*w*) instead of 10% *w*/*v* Na_2_SO_3_ was added to the blend before heat pressing (Elastic modulus: 56.36 ± 5.64 N/mm^2^, stress at fracture: 0.55 ± 0.12 N/mm^2^).

The influence of SDS, urea, and guanidinium chloride, which are commonly employed denaturing agents for proteins, on the mechanical properties of RM-based materials was investigated. The Young’s modulus of the obtained slabs decreased by two orders of magnitude compared to the unmodified RM material, indicating the important role of the ordered structure on mechanical strength. However, the stress at fracture remained relatively constant and small, suggesting that the weak points remain the same (Figure 1 and Table 5). Urea destabilizes globular proteins by establishing robust hydrogen bonds with water molecules that surround the protein. Simultaneously, it interferes with the hydrogen bonds within the protein structure, leading to the formation of partially unfolded protein conformations and flexible peptide chains. Similarly, the interaction with SDS alters both tertiary and quaternary protein structures by disrupting the hydrophobic and electrostatic interactions within the protein without breaking covalent bonds. This results in the protein structure becoming partially unfolded and more flexible. The elongation remains relatively low due to fractures occurring in defective areas, which cause the material to break at the interface between distinct domains.

The above results suggest that a higher degree of denaturation and, consequently, a greater number of cross-links are achieved when a reducing agent such as Na_2_SO_3_ acts upon covalent S-S bonds, compared to the utilization of denaturing agents like urea, guanidinium chloride, and SDS.

As shown in Table 6, when both Na_2_SO_3_ and guanidinium chloride are employed in the production of RM-based materials, an increase in stress at fracture and in the elastic modulus was observed. Denaturation exposes the hydrophobic residues that are buried within the core of folded protein molecules at the molecular surface. Moreover, these hydrophobic groups are capable of engaging in more extensive hydrophobic interactions among protein molecules during the compression molding process [47,48]. Young’s modulus increases significantly due to the formation of cross-links between the protein chains and the cross-linker (Epoxy-PEG) due to the higher number of reactive groups exposed by the denaturing agent. Moreover, the plasticizer effect of water was observed when RM-R2D2 7% and RM-R2D2 14% were compared, as evidenced by the reduction in Young’s modulus and tensile stress. Indeed, water has a high dielectric constant and establishes strong interactions with the hydrophilic and charged groups of the proteins through the formation of hydrogen bonds [49].

### 3.3. Thermal Properties

**Figure 2:**
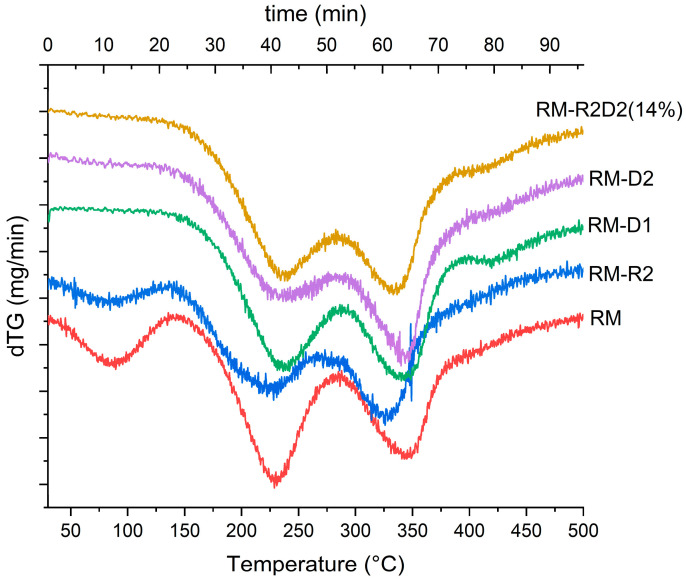
First-derivative curve of TGA of rapeseed meal protein-based materials, RM, RM-R2, RM-D1, RM-D2, and RM-R2D2 14%.

To determine the effect of the different compositions on the thermostability of the material, we performed the thermogravimetric analysis of all the samples. The TGA and first-derivative curves (Figure S1 and Figure 2, respectively) show a weight loss profile for all materials, similar to what observed for other protein-based materials [47]. The initial weight loss, between 30 °C and ca 150 °C, is particularly evident in samples RM and RM-R2 (Figure 2) and corresponds to a loss of moisture. In particular, RM exhibits a higher amount of residual and eliminable moisture, which is therefore considered non-structural. In contrast, the other specimens did not display any water elimination, indicating their inability to absorb water due to increased reticulation or a higher structural water content. Additionally, the RM-D2 and RM-R2D2 samples, which have a greater reticulation degree, show an inversion of the area ratios between the first and second thermal degradation steps, reflecting a decline in quantity. The peak of the first degradation step (around 225 °C) shifts slightly to a higher temperature, suggesting a marginal increase in stability. The second stage (ca 150 °C–250 °C) can be attributed to the loss of glycerol (bp 290 °C). A third step weight loss from 250 °C to ca 400 °C is due to the degradation of residual protein-rich material. RM-D1 also shows an extra stage (400 °C–500 °C), probably due to the decomposition of urea with the loss of ammonia and carbon dioxide.

A slight shift to higher temperatures in the weight loss of glycerol can be observed going from RM to RM-R2D2 14%, except for RM-R2. That indicates an enhanced thermal stability of the material containing both the reducing and the denaturing agent (RM-R2D2 14%).

### 3.4. Protein Conformation Analysis

**Figure 3:**
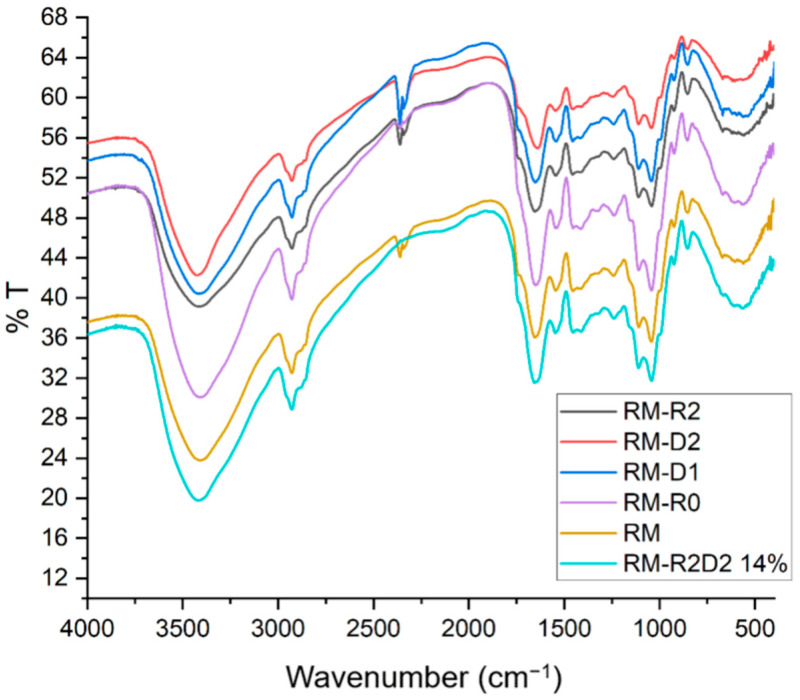
Overlapped full FT-IR spectra of rapeseed-meal-based materials.

**Figure 4:**
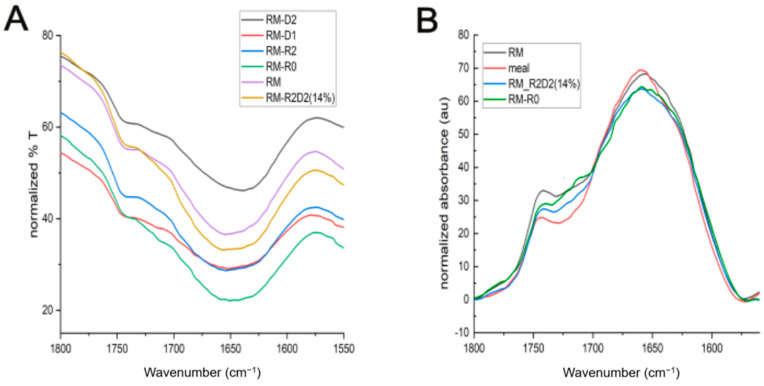
FT-IR spectra of amide I band of: (**A**) RM-R0, RM, RM-R2, RM-D1, RM-D2, and RM-R2D2 14% materials. (**B**) Rapeseed meal, RM-R0, RM, and RM-R2D2 14% materials (baseline corrected).

Untreated meal and the modified materials were analyzed by FT-IR spectroscopy. No substantial change can be observed when comparing the full spectra of the materials (Figure 3). To investigate eventual changes in the secondary structures of the protein components, we compared the amide I band region (from ca 1590 to 1700 cm^−1^), which is mainly due to the stretching vibration mode of the carbonyl in the amide groups and is representative of protein secondary structures (Figure 4).

The amide I bands of RM-R, RM-R2, RM-D1, RM-D2, and RM-R2D2 14% show slight differences in the range of 1625–1700 cm^−1^ that are more evident in the case of RM-D2 (Figure 4A). However, comparing the amide I band in the spectrum of the meal with the one in the spectra of RM, RM-R0, and RM-R2D2 14% (Figure 4B), a change in shape in the range of 1600–1625 cm^−1^ can be observed. This can be ascribed to the contribution of H-bonded β-sheets because of protein aggregation [49,50], as shown in the spectra reported in Figure S2. A notable difference was observed in the band areas of the new materials compared to the raw rapeseed meal, highlighting the impact of thermal treatment on secondary structures. Specifically, differences are significant at 1622–1624 cm^−1^, and can be attributed to the contribution of strongly hydrogen-bonded β-sheets (Table 7) [49,51]. Aggregation could also be ascribed to the isopeptide bond formation [52] that can be formed at high temperatures, mainly due to the presence of lysine and tyrosine residues (5.55% and 3.22%, respectively, present in the crude protein-based material).

Moreover, the higher contribution of the peak at 1745 cm^−1^ to the amide I band in the spectra indicates the formation of the desired cross-links through inter- or intra-molecular ester bonds between the Asp and Glu sidechains in the protein and in the lignin components of the meal and the epoxide [49,51].

### 3.5. Material Appearance

All the material obtained from compression molding were brown, compact and flexible.

Scanning electron microscopy was used to investigate the morphology of the material in more detail. When the materials were fractured at room temperature, it was possible to observe a clear change in their morphology, comparing both the section and the interior of the single specimens to each other (Figures S3 and S4). Indeed, the presence of casein and the crosslinker (comparison RM-R0/RM) gives a more compact aspect to the section of the material (Figure S3): it is still possible to distinguish layers in RM, but they are much less separated than in the case of R0. When 20% Na_2_SO_3_ is added (specimen RM-R2), the material appears more homogeneous. Different morphologies can be observed in the case of RM-D1 and RM-D2, probably because of the different effects of the denaturing agent used. Among all the specimens analyzed, RM-R2D2 14% is the one that looks the most compact and uniform. By taking images at a higher magnification (Figures S5–S8), it was possible to distinguish the cells of rapeseed: a well-defined cell wall was visible in RM-R0, while cells were more broken in the other specimens, which indicates that the high protein content in them disappeared and was more available for processing.

**Figure 5:**
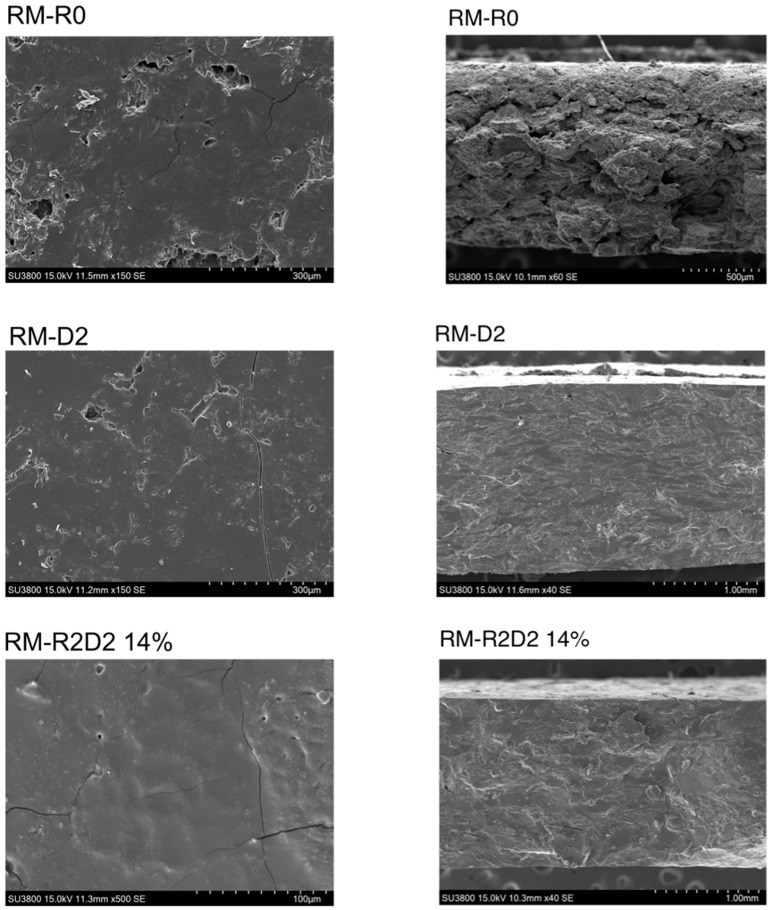
Scanning electron microscopy images of surface (**left**) and cryofracture cross-section (**right**) of specimens RM-R0, RM-D2, and RM-R2D2 14%.

We also performed SEM measurements on cryogenic fractured RM-R0, RM-D2, and RM-R2D2 14% (Figure 5). In this case, the differences in the sections are striking: while RM-R0 is clearly still a simply pressed material, RM-D2 and RM-R2D2 14% are homogeneous and compact materials.

## 4. Conclusions

In this study, raw rapeseed meal was processed with appropriate interactive additives by pression molding to obtain a novel protein-rich material. Moreover, a series of investigations were performed to understand the effect of several treatments and additives on the final properties. The addition of Rennet casein increased the protein content of the material and clearly improved its mechanical resistance. Different reducing and denaturing agents were tested at various concentrations to induce protein denaturation and to understand the role of the molecular feature on the material’s ultimate properties. The polypeptide chain became more flexible and, at the same time, led to functional groups that were more accessible for the crosslinking reaction. The best results were obtained when a 20% *w*/*w* aqueous solution of Na_2_SO_3_, acting both as a disulfide-bond-reducing agent and as plasticizer, and 1 M guanidinium chloride, acting as a denaturing agent, were added to the blend (14%, *w*/*w* total), with a significant increase in Young’s modulus and very modest elongation at fracture. The study described up to now is certainly innovative and informative, despite the mechanical properties not yet being industrially competitive. However, our results suggest a promising pathway for developing a protein-rich material for future industrial application. A change in the protein structure was evidenced by the FT-IR analysis when comparing the amide I bands of the specimens with different compositions to each other and to the one comprising rapeseed meal, indicating effective protein denaturation. The presence of disulfide-bond-reducing and denaturing agents caused the partial unfolding of the proteins and led to a major exposure of the amino acid side chains of the protein. Thus, hydrophobic groups are capable of engaging in more extensive hydrophobic interactions among amino acids in the protein molecules and the reactive functional groups form more cross-links with the crosslinker. Moreover, TGA revealed the improved thermal stability of the material containing both the reducing and the denaturing agent (RM-R2D2 14%), suggesting the stronger interaction of glycerol with the other component of the material, particularly with the amino acid side chains of the denatured proteins. Taken together, the presented results demonstrate the potential use of cross-linked, rapeseed protein-rich meal-based materials obtained by pression molding as an alternative to environmentally demanding polymeric materials.

## Figures and Tables

**Table 1:** Examples of feedstock and processing used to produce protein-based biomaterial.

Feedstock	Processing	Reference
Albumen protein isolated and crayfish flour	Injection moulding	[23]
Defatted Soybean meal	Casting	[28]
Gluten meal and Wood fiber	Extrusion	[31]
Crambe and Carinata Oilseed Meal	Compression moulding	[23]
Rapeseed meal	Compression moulding	[19]
Rapeseed Meal, Fruit Pomace and Microcrystalline Cellulose	Compression moulding	[33]
Red and White Wine Grape Pomaces	Compression moulding	[30]
Rapeseed Protein Isolates	Casting	[34]
Rapeseed Protein Isolates	Casting	[35]
Rapeseed Protein Isolates	Casting	[36]

**Table 2:** Rapeseed meal-based (RM) specimen formulation.

Sample	Formulation			
	Rapeseed Meal/Compatibilizer	Glycerol	Water Solution	Epoxy-PEG
RM-R0	100:0	27%	Distilled water 7%	-
RM	04:01	27%	Distilled water 7%	2× mol
RM-R1	04:01	27%	(Na_2_SO_3_ 10%) 7%	2× mol
RM-R2	04:01	27%	(Na_2_SO_3_ 20%) 7%	2× mol
RM-D1	04:01	27%	(UREA 2 M) 7%	2× mol
RM-D2	04:01	27%	(Guanidine 1 M) 7%	2× mol
RM-D3	04:01	27%	(SDS 3%) 7%	2× mol
RM-R1D2 (7%)	04:01	27%	(Na_2_SO_3_ 10% + Guanidine 1 M) 7%	2× mol
RM-R1D2 (14%)	04:01	27%	(Na_2_SO_3_ 10% + Guanidine 1 M) 14%	2× mol
RM-R2D2 (7%)	04:01	27%	(Na_2_SO_3_ 20% + Guanidine 1 M) 7%	2× mol
RM-R2D2 (14%)	04:01	27%	(Na_2_SO_3_ 20% + Guanidine 1 M) 14%	2× mol

**Table 3:** Tensile mechanical properties of blends with different crosslinkers.

Sample	Mechanical Properties		
	Stress at Fracture (N/mm^2^)	Elastic Modulus (N/mm^2^)	Elongation at Fracture (%)
RM	0.86 ± 0.24	172.23 ± 2.17	0.83 ± 0.11
RM-ES	0.38 ± 0.06	55.91 ± 9.93	2.18 ± 0.25
RM-NE	0.81 ± 0.02	60.94 ± 0.85	4.70 ± 0.56

**Table 4:** Tensile mechanical properties of blends with different concentrations of reducing agent (Na_2_SO_3_).

Sample	Mechanical Properties		
	Stress at Fracture (N/mm^2^)	Elastic Modulus (N/mm^2^)	Elongation at Fracture (%)
RM	0.86 ± 0.24	172.23 ± 2.17	0.83 ± 0.11
RM-R1	2.00 ± 0.37	234.82 ± 8.15	1.24 ± 0.37
RM-R2	1.13 ± 0.06	103.60 ± 8.66	1.92 ± 0.06

**Table 5:** Tensile mechanical properties of blends containing denaturing agents (Urea, guanidinium chloride, Sodium Dodecyl Sulphate (SDS)).

Sample	Mechanical Properties		
	Stress at Fracture (N/mm^2^)	Elastic Modulus (N/mm^2^)	Elongation at Fracture (%)
RM	0.86 ± 0.24	172.23 ± 2.17	0.83 ± 0.11
RM-D1	0.90 ± 0.22	75.67 ± 2.00	1.96 ± 0.35
RM-D2	0.82 ± 0.19	95.17 ± 0.10	1.70 ± 0.33
RM-D3	0.59 ± 0.15	70.35 ± 1.77	1.49 ± 0.33

**Table 6:** Tensile mechanical properties of blends containing sodium sulfite and guanidinium chloride in different percentages.

Sample	Mechanical Properties		
	Stress at Fracture (N/mm^2^)	Elastic Modulus (N/mm^2^)	Elongation at Fracture (%)
RM-R1D2 7%	0.85 ± 0.13	214.18 ± 4.27	0.88 ± 0.08
RM-R1D2 14%	1.24 ± 0.20	216.89 ± 0.98	1.20 ± 0.06
RM-R2D2 7%	1.87 ± 0.34	277.99 ± 1.95	1.38 ± 0.27
RM-R2D2 14%	1.53 ± 0.20	257.71 ± 9.40	0.18 ± 0.28

**Table 7:** Area (%) of the resolved peaks (Figure S2) present in the amide I region of the rapeseed meal and of the materials RM, RM-R0, and RM-R2D2 14%.

Peak (ῦ, cm^−1^)	A% Rapeseed Meal	A% RM	A% RM-R0	A% RM-R2D2 14%
1622–1624	10.4	28.3	22.21	17.4
1662–1665	77.0	30.5	60.39	58.1
1711–1729	12.3	39.6	15.54	22.2
1745	0.3	1.6	1.86	2.4

## Data Availability

No existing datasets or raw data have been analysed in the manuscript.

## Supplementary Materials

The following supporting information can be downloaded at: https://www.mdpi.com/article/10.3390/biom14080982/s1, Figure S1: TG curves of specimens; Figure S2: Amide I band in the FT-IR spectra of the materials: (A) meal, (B) RM-R0, (C) R0, and (D) RM-R2D2 14%; Figure S3: SEM of the rapeseed meal-based materials (cross section, room temperature) at 500 or 300 μm; Figure S4: SEM of the rapeseed meal-based materials (longitudinal section, room temperature); Figure S5: SEM of the rapeseed meal-based materials (cross section, room temperature) at 100 μm, Figure S6: SEM of the rapeseed meal-based materials (longitudinal section, room temperature) RM-R0, RM and RM-R2; Figure S7: SEM of the rapeseed meal-based materials (longitudinal section, room temperature) D1 and D2; Figure S8: SEM of the rapeseed meal-based materials (longitudinal section, room temperature) RM-R2D2 14%.
