# Supplementary material for: Study of the Preparation and Properties of Chemically Modified Materials Based on Rapeseed Meal
Source: Biomolecules. 2024 Aug 10;14(8):982. doi: 10.3390/biom14080982 (PMC11352606; doi:10.3390/biom14080982)
Supplement: Supplementary file 1 [file biomolecules-14-00982-s001.zip › biomolecules-3099787-supplementary.pdf]

SUPPLEMENTARY INFORMATION

Study of the preparation and properties of chemically modified materials based on rapeseed meal

Sara Aquilia,[1,2,3] Luca Rosi,[2] Michele Pinna,[3] Sabrina Bianchi,[3] Walter Giurlani,[2] Marco Bonechi,[2] Francesco Ciardelli,[3*] Anna Maria Papini,[1,2*] Claudia Bello[1,2]

1. Interdepartmental Research Unit of Peptide and Protein Chemistry and Biology, University of Florence, Via della Lastruccia 13, I-50019 Sesto Fiorentino, Italy
2. Department of Chemistry "Ugo Schiff", University of Florence, Via della Lastruccia 13, I-50019 Sesto Fiorentino, Italy
3. Spin-PET S.r.l., Viale R. Piaggio 32, I-56025 Pontedera, Italy

* Correspondence: annamaria.papini@unifi.it; ciardelli@spinpet

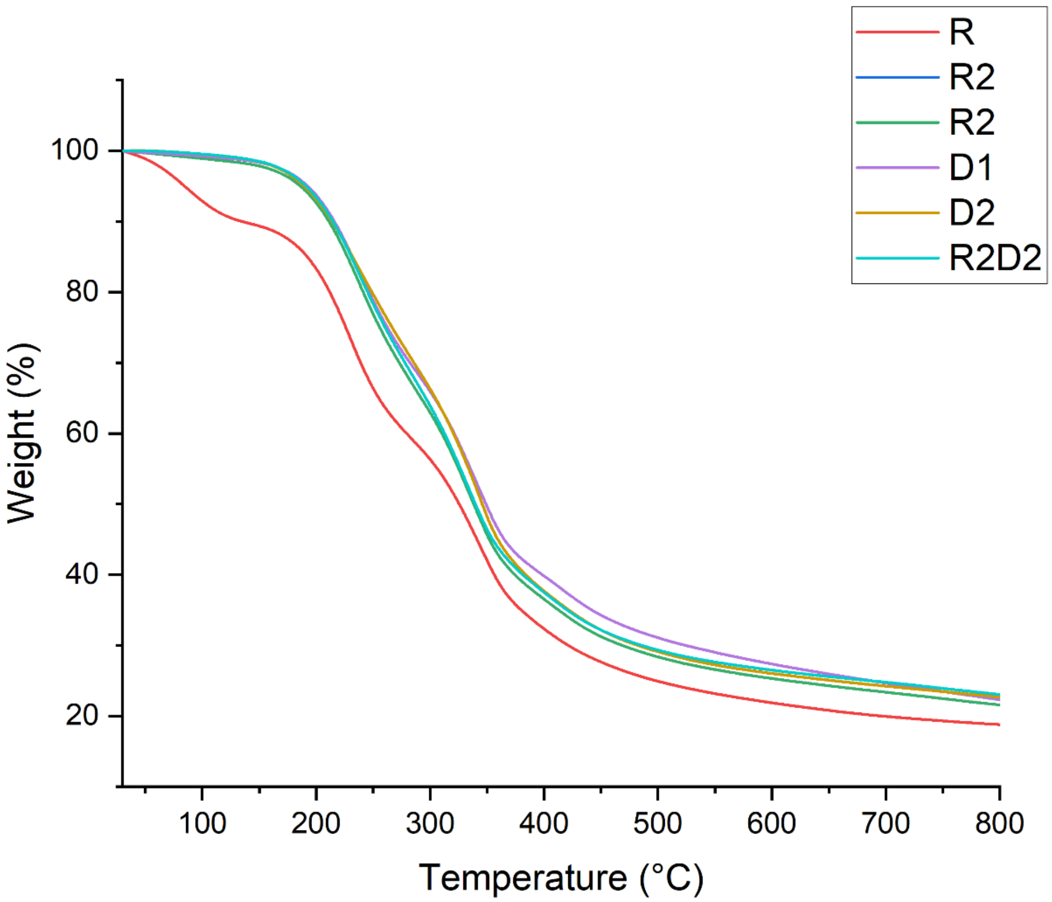

Figure S1. TG curves of specimens RM, RM-R2, RM-D1, RM-D2 and RM-R2D2 14%.

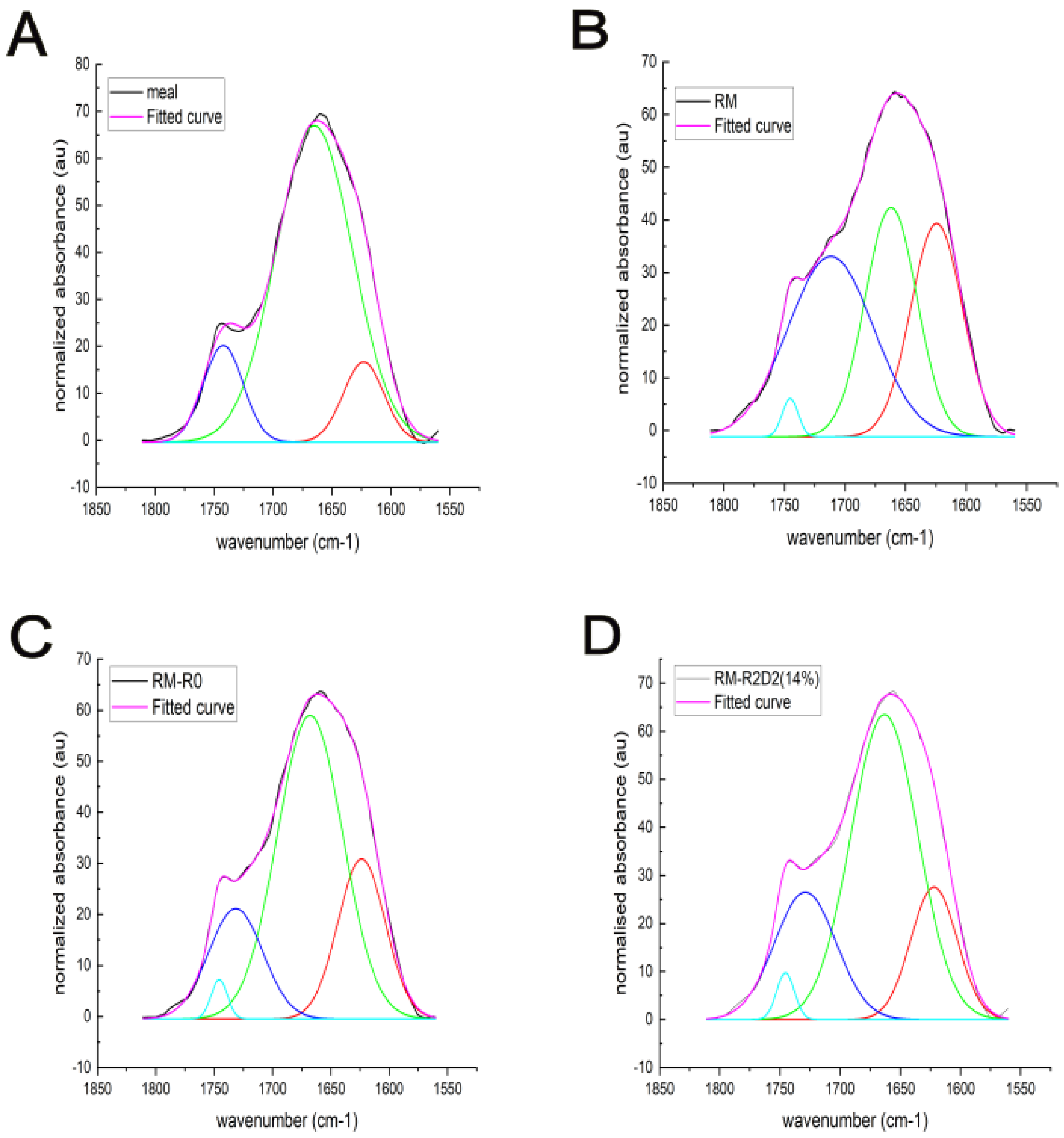

Figure S2. Amide I band in the FT-IR spectra of the materials: (A) meal, (B) RM-R0, (C) R0, and (D) RM-R2D2 14%.

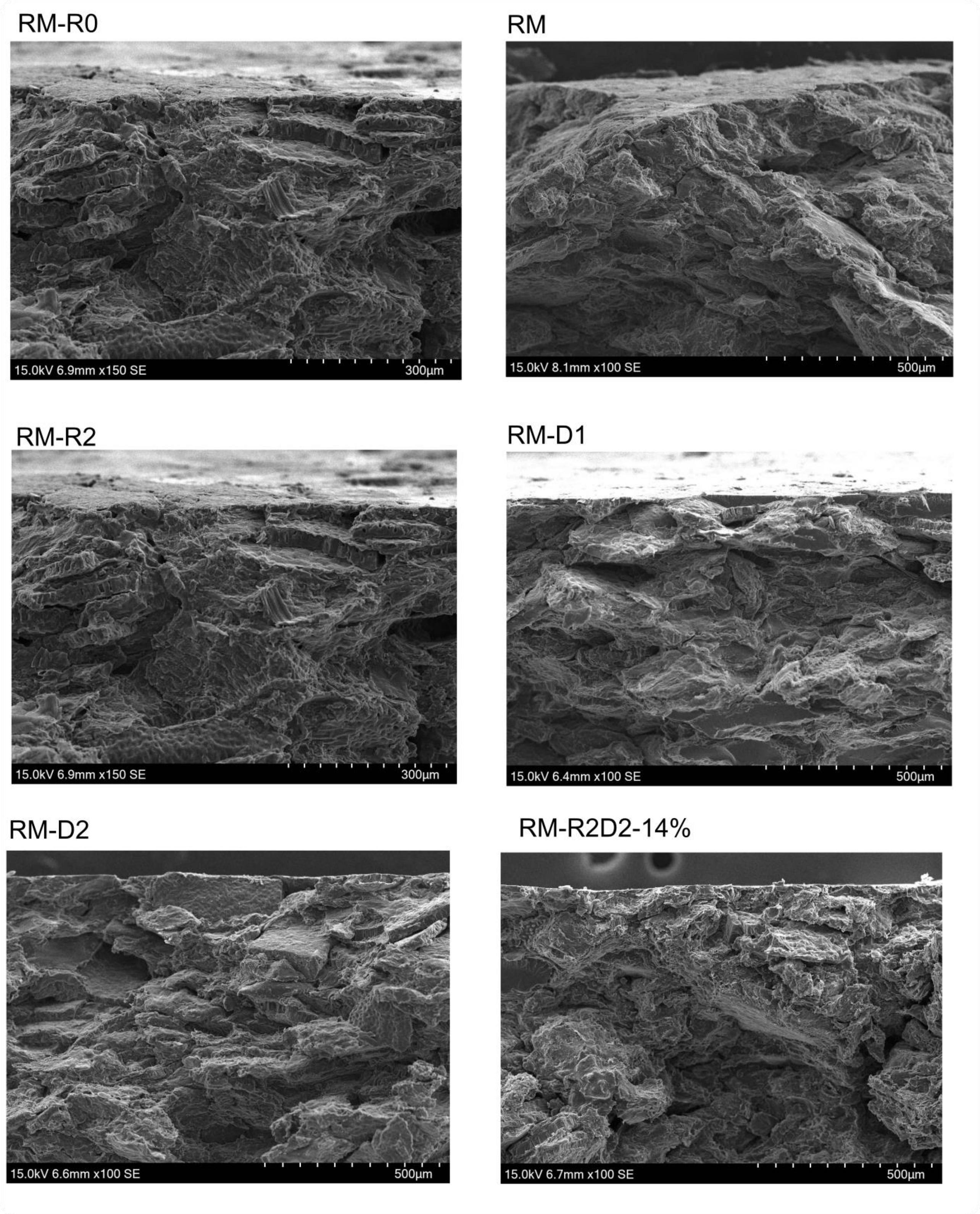

Figure S3. SEM of the rapeseed meal-based materials (cross section, room temperature) RM-R0, RM, RM-R2, RM-D1, RM-D2, RM-R2D2 14% at 300μm or 500μm.

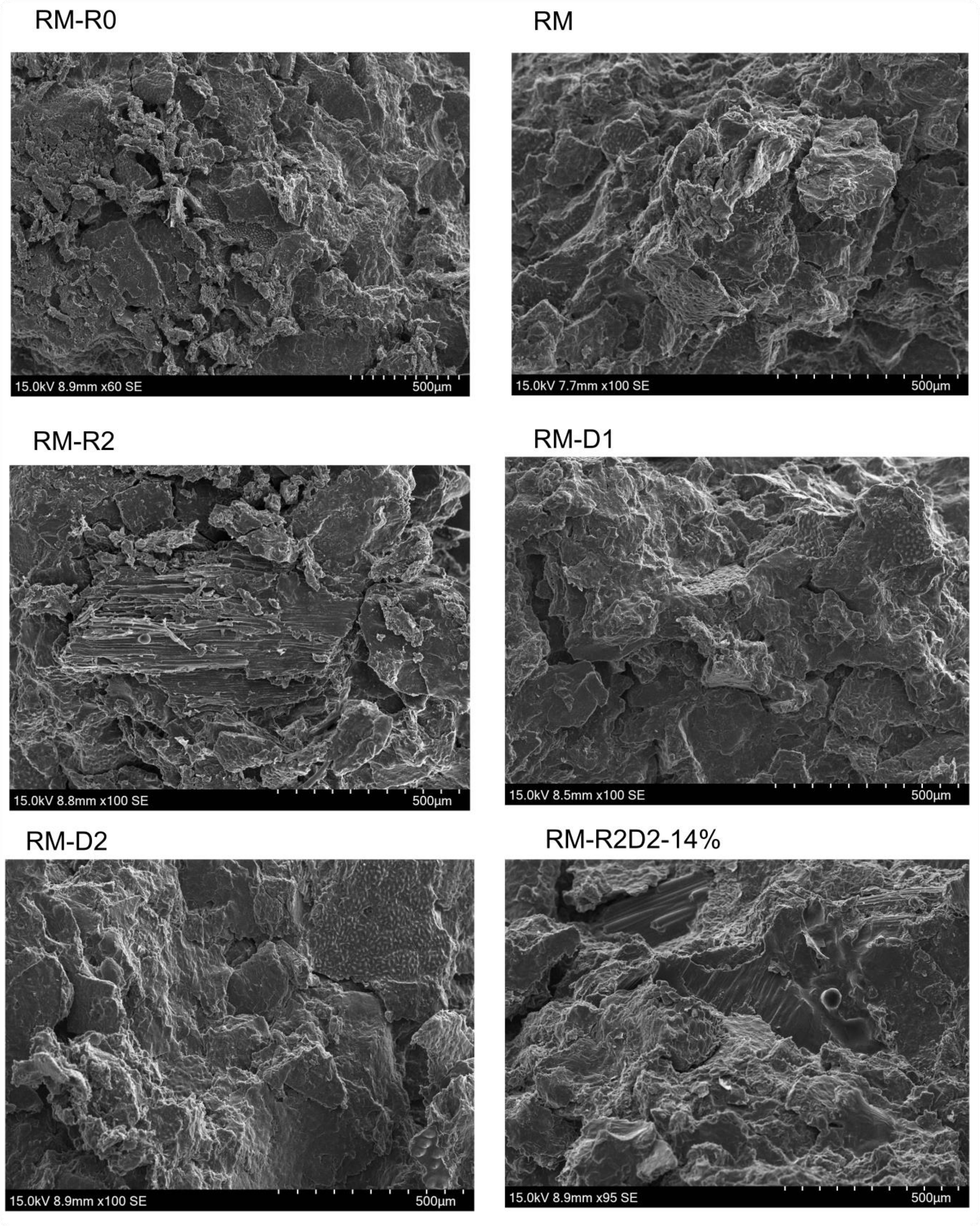

Figure S4. SEM of the rapeseed meal-based materials (longitudinal section, room temperature) RM-R0, RM, RM-R2, RM-D1, RM-D2, RM-R2D2 14% at 500µm.

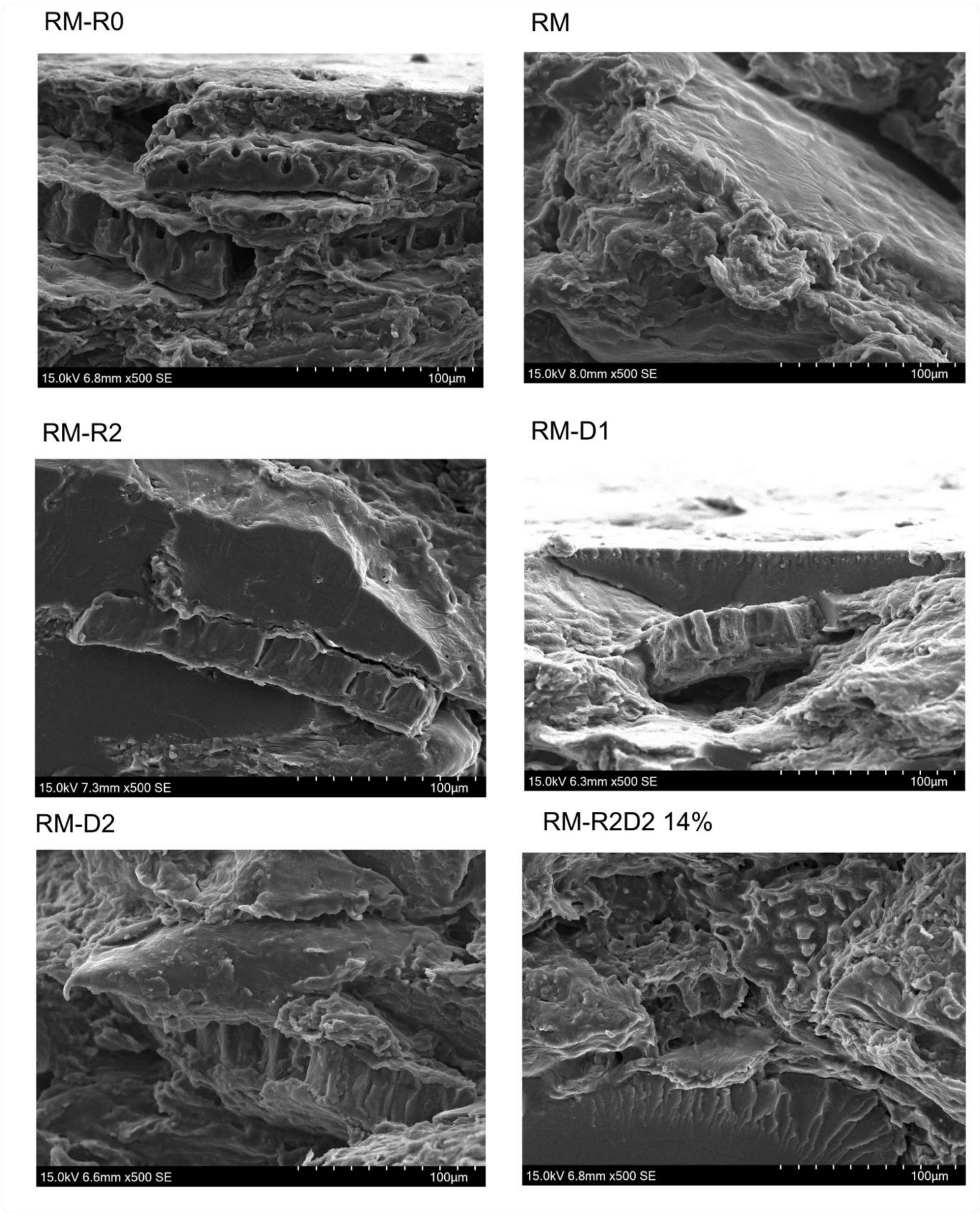

Figure S5. SEM of the rapeseed meal-based materials (cross section, room temperature) RM-R0, RM, RM-R2, RM-D1, RM-D2, RM-R2D2 14% at 100µm.

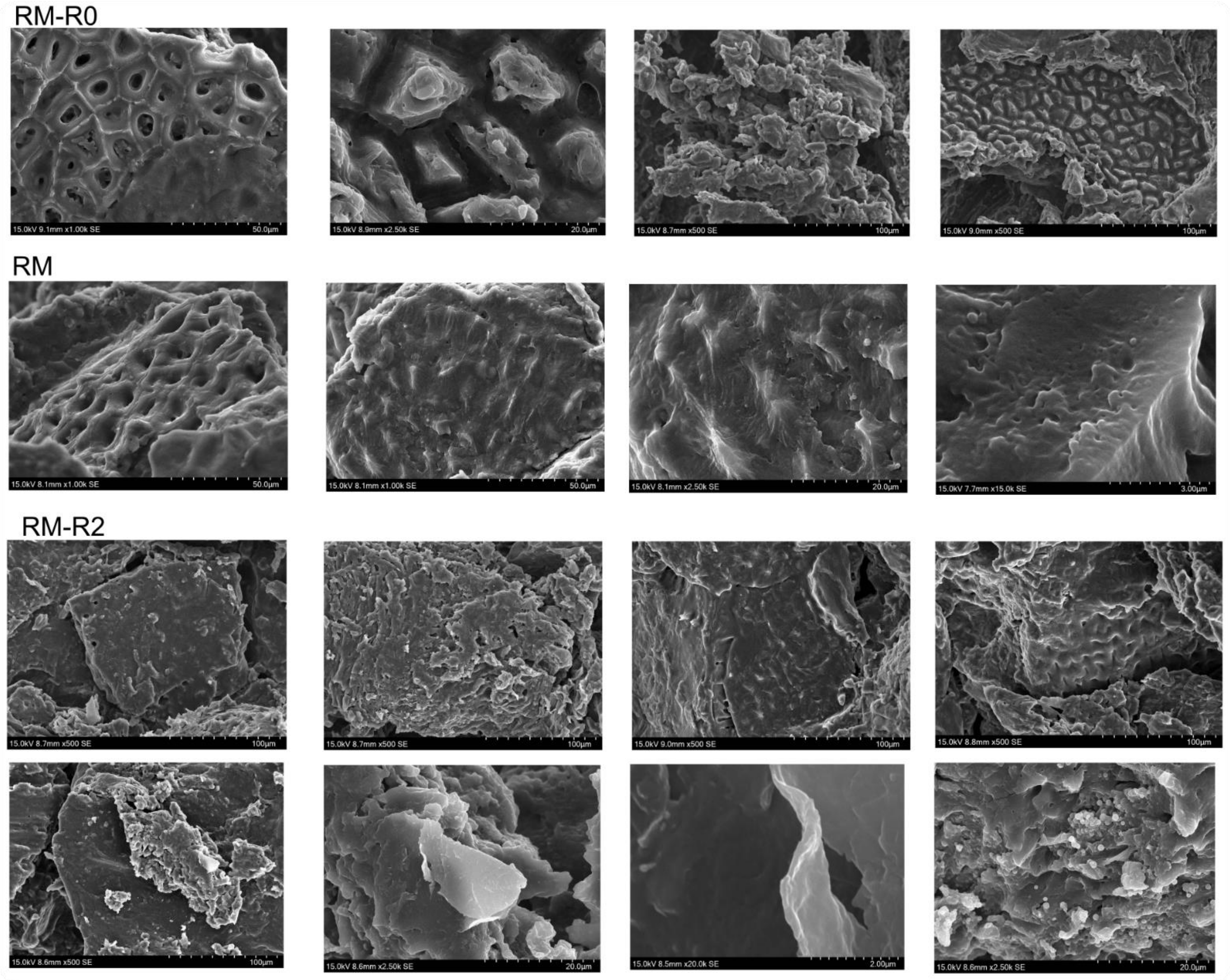

Figure S6. SEM of the rapeseed meal-based materials (longitudinal section, room temperature) RM-R0, RM and RM-R2 at 100um to 2μm.

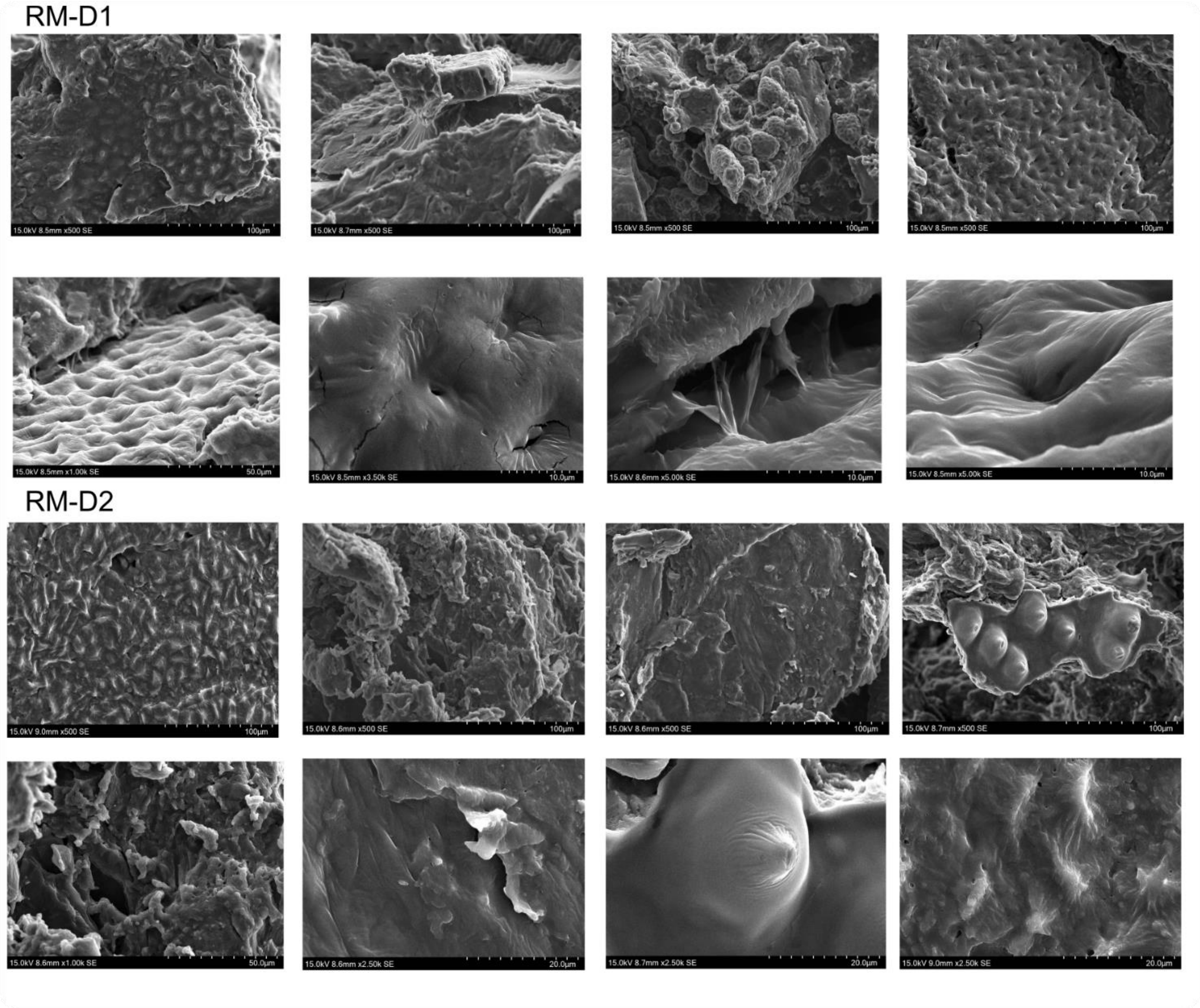

Figure S7. SEM of the rapeseed meal-based materials (longitudinal section, room temperature) D1 and D2 at 100um to 10um.

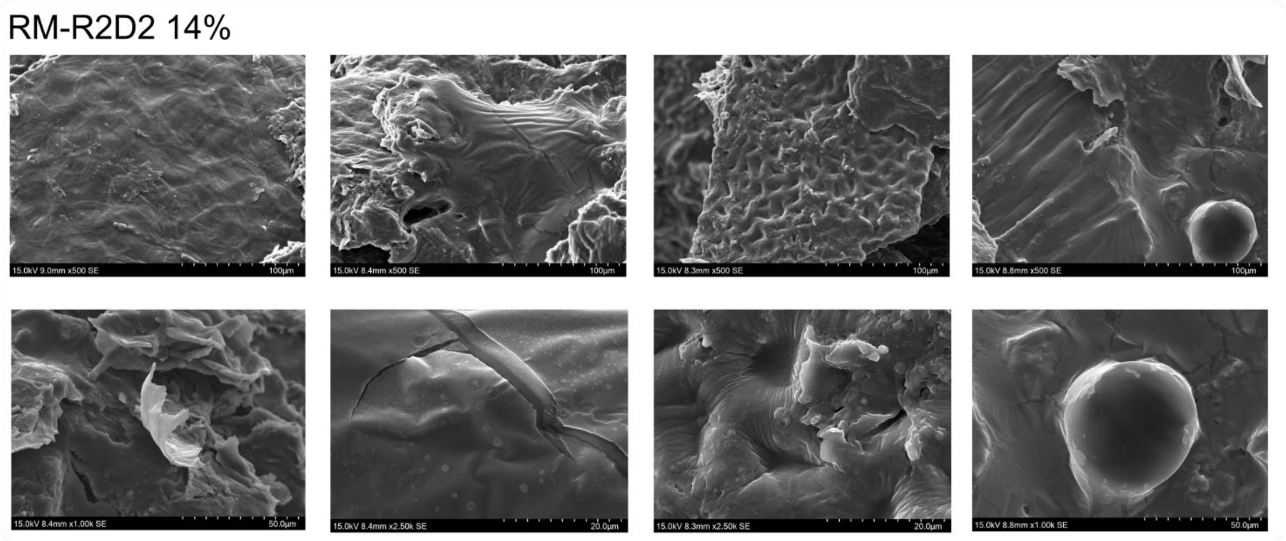

Figure S8. SEM of the rapeseed meal-based materials (longitudinal section, room temperature) RM-R2D2 14% at 100um to 20um.
